# Assessing and monitoring clinical practice of undergraduate nursing students: a middle eastern context

**DOI:** 10.3389/fmed.2025.1524230

**Published:** 2025-02-03

**Authors:** Shehnaaz Mohamed, Nganga Sinnasamy, Sumayya Ansar, Meagan LaRiviere

**Affiliations:** Faculty of Nursing, University of Calgary in Qatar, Doha, Qatar

**Keywords:** nursing student, clinical assessment tool, formative assessment, clinical evaluation, clinical skills assessment, clinical competence, undergraduate nursing students, clinical nursing education

## Abstract

This paper presents an innovative Weekly Clinical Skills Progress (WCSP) tool to support the assessment of undergraduate nursing students in their clinical placements. The WCSP tool was implemented at the University of Calgary in Qatar (UCQ) Nursing Program in Spring 2024 to address inconsistencies in assessment documentation related to the absence of clearly defined proficiency levels in clinical courses. The UCQ clinical faculty trialed the newly developed WCSP tool on eighty-seven third-year nursing students enrolled in the clinical course Nursing Practice for High Acuity and Chronic Conditions. These students were divided into 11 groups, each consisting of six to seven members per instructor, and were placed in various medical-surgical clinical sites throughout Hamad Medical Corporation (HMC) in Qatar. During the course implementation and following, feedback from faculty, students and buddy nurses indicated the WCSP tool clarified the clinical goals, enabled consensus on clinical proficiency levels according to the course outline, and assessments were more consistent. Though the WCSP tool is still being refined, and more qualitative and quantitative research is needed, this paper contributes valuable preliminary results and recommendations that benefit nursing programs worldwide.

## 1 Introduction and background

Teaching and learning in clinical nursing settings involves situational learning that is dynamic and often unpredictable. This environment enables students to apply theoretical knowledge and engage in complex reasoning. Regular formative assessments, monitoring, and feedback in clinical practice are crucial as they provide nursing students with a clear pathway to achieving their entry-to-practice competencies (1). Despite the critical role of clinical assessment in the nursing curriculum, monitoring student progression in clinical settings has been challenging for clinical instructors at UCQ. The faculty instructors were using an assessment template that lacked clearly defined markers of clinical learning and guides for assessing those markers. The problem with the template became apparent when the final clinical documentation showed various methods and styles and a wide range of depth and breadth of assessment. The dynamic nature of clinical experiences and the diverse clinical faculty perspectives on assessment contributed to the documentation inconsistencies. These issues with clinical assessment documentation led to gaps and confusion in evaluating students’ overall progress across clinical courses.

Assessment of nursing students’ competence in the clinical setting is complicated by a lack of global consensus on how to measure clinical skills. The literature reveals significant knowledge gaps regarding the methods educators use to monitor students in these environments, emphasizing the need to improve the clinical guidance skills of healthcare professionals (2). Although nursing is a universal profession with common goals, the literature shows that each country and educational institution follows its own method of assessing clinical competencies (3). The complexity of assessing students in clinical practice stems from the dynamic nature of the clinical environment and the specific clinical proficiency expected in each course and overall curriculum. Evaluating learning in clinical settings is more challenging compared to theoretical knowledge or simulation labs, where assessments occur in controlled conditions. This complexity has long been acknowledged, with ongoing efforts to develop clear and understandable assessment forms for both students and instructors (1, 4, 5). Despite identifying these challenges, there remains a scarcity of universally applicable solutions to address the issues surrounding clinical assessments.

At UCQ, the vague consensus on clinical skills measurement was epitomized by the use of broad, generalized criteria in clinical assessment. Clinical skills were assessed according to the three categories of basic, intermediary and proficient, terms used in all 4 years of the nursing program. These indiscrete, broad terms forced assessors to rely heavily on subjective judgment that did not support student progression. The reliance on subjectivity made it challenging to analyze clinical assessment results over the 4 years of learning and ultimately evaluate diverse student competencies for graduation (6). The lack of clear and objective tools for measuring competencies in clinical settings worsened these difficulties since the tools contained these three vague categories of skills competencies. Zasadny and Bull (7), found that competency measurement remains ambiguous and subjective, with interpretations varying widely. A study by Redfern et al. (8) discussed that while various tools have been developed, there are still concerns regarding their reliability and validity.

Numerous assessment tools are utilized globally to evaluate nursing students’ performance in clinical settings. These tools vary significantly in their design, scope, and application, each aiming to measure different aspects of clinical competence, such as practical skills, critical thinking, and professional behavior. They range from structured skills checklists and Objective Structured Clinical Examinations (OSCEs) to competency-based assessments using checklists. A systematic review of reviews by Immonen et al. (9) emphasized the need for a structured approach to assessing students and the authors highlighted the importance of using valid and reliable tools. In addition, it is essential to support buddy nurses (staff nurses partnered with students in clinical sites), who are responsible for student evaluations by ensuring they are adequately trained in using these tools (9, 10). Any difference of expectations between a buddy nurse, student and clinical instructor creates conflicts in goal setting and students achieving competencies realistically (11).

At UCQ, the undergraduate nursing program utilizes a concept-based approach (CBA), which is designed to foster a deep and comprehensive understanding of fundamental nursing concepts among students. This pedagogical method emphasizes critical thinking and the application of knowledge in varied and complex healthcare environments. As such, there is a critical need to synergize concepts in theory courses with competencies in clinical courses. A comprehensive grasp of nursing concepts is essential for nurses, who are often required to make informed, evidence-based decisions in unpredictable and fast-paced clinical settings (12). For the assessment of clinical competencies, UCQ aligns with the College of Registered Nurses of Alberta (CRNA)’s entry-level competencies (ELC) for registered nurses. These competencies serve as the standard for evaluating students’ readiness for professional practice, specifically in Alberta, Canada. However, several challenges have been noted in the application of this framework within the local context in Qatar. The CRNA ELC document provides a simplistic list of competencies not amenable to curricular scaffolding, which makes planning for progressive, discrete learning and assessment difficult. Further, some of the Canadian-based terminology is challenging for students in Qatar where English is not their first language. The English terminology has been a barrier for students to comprehend the ELCs to their full scope. Likewise, certain Canadian-context competencies are not applicable in Qatar since they do not fully apply to the cultural and clinical setting in which UCQ students are trained. Examples of competencies not applicable in Qatar are ELC 6.1 “Acquires knowledge of the *Calls to Action of the Truth and Reconciliation Commission of Canada*,” and ELC 7.3 “Advocates the use of Indigenous health knowledge and healing practices in collaboration with Indigenous healers and Elders consistent with Calls to Action of the Truth and Reconciliation Commission of Canada” (13). These two ELCs are clearly culturally tied to the Canadian context. These challenges highlighted the need for adaptations to ensure that the competencies are both accessible and relevant to the students’ educational and clinical environment. The CRNA competencies were used as a framework for the UCQ Canadian nursing curriculum, while the Qatar nursing competencies were mapped into the course outlines following implementation. The WCSP tool aimed to find competency language that offered more synergy and clarity for both the faculty and stakeholders.

Effective teaching strategies rely on continuous evaluation to enhance student learning outcomes. In concept-based education, the approach shifts the focus from memorizing facts to understanding overarching principles and ideas. Formative assessments lay the groundwork by identifying and addressing gaps, thereby reinforcing concepts throughout the course (14). In clinical education, formative assessment promotes concept understanding by facilitating students’ gradual development of knowledge, skills and attitudes throughout their clinical learning experience. Students are supported in their progression by increasing lower-stakes assessments, allowing them to prepare for summative assessments before the course ends. Similarly, clinical faculty may readily identify students at risk of failing and plan timely learning interventions. By reinforcing good practices and motivating students to meet the required competency levels, formative feedback significantly contributes to student learning in clinical settings (14). Similarly, Gaberson et al. (15) emphasized that “for clinical evaluation to be effective, the teacher should provide continuous feedback to students about their performance and how they can improve it.” Without ongoing feedback, students may assume their performance is satisfactory, which can prevent them from recognizing areas needing improvement (14, 15). Continuous feedback is essential for guiding students, helping them to refine their skills, and ensuring they make the necessary adjustments to meet learning objectives effectively. Both formative and summative assessments play a role in ensuring students can integrate and apply concepts effectively (16).

To ensure meaningful assessments, they must be deeply rooted in the realities of clinical placements and aligned with clear and objective criteria (17). This requires a consistent framework which can reflect the actual demands and expectations of clinical environments, allowing students to be assessed on skills and competencies they will use in practice. A review by Lewallen and Van Horn (18) of 88 papers on clinical evaluation in nursing education revealed a lack of a standardized definition of clinical competence. Similarly, Liou et al. (19) emphasized the need to revise institutional guidelines for nursing education and clinical practice to enhance nursing skills and critical thinking and address the existing challenges in clinical practice. At the instructional level, the lack of specific guidelines that align clinical competency proficiency with students’ academic progression further complicates the evaluation process. Vague proficiency assessment, according to the terms basic, intermediary and proficient, makes it difficult for students to understand how their clinical assessments reflect their overall growth, resulting in inconsistent learning outcomes.

Discussions with instructors, along with the authors’ personal experiences, revealed a lack of consensus between clinical instructors on how weekly goals can be set to ensure students meet nursing competencies, according to CRNA. While most instructors were committed to ensuring nursing students have a robust and dynamic clinical experience, they often encountered challenges with the evaluation tools provided, which may not be fully understood or utilized effectively. Conversely, while students appreciated support in clinical settings, they have reported inconsistencies in expectations from clinical instructors, often feeling confused due to varying messaging. This paper presents the WCSP tool created in response to the challenges reported by both students and clinical instructors. The WCSP tool integrates concept and competency assessment with the scaffolding of essential skills, providing a structured week-by-week framework for medical-surgical clinical placements. The tool also breaks down vague terminology (beginner, intermediate and proficient), to offer clear, specific expectations for each week. The WCSP tool addressed the issues highlighted in the literature, such as the lack of detailed guidance on the competencies students are expected to achieve progressively throughout the clinical placement.

## 2 Methodology

To address the identified gaps in knowledge and inconsistencies in how clinical instructors monitor and assess student groups across various clinical settings, we employed an action research design. Action research is appropriate to the development and implementation of a novel clinical evaluation tool such as WCSP because this is a critical reflection and explanation of our practice in clinical assessment (6). The classic characteristic of action research is to respond to a problem by co-planning, designing, implementing, observing and reflecting on the solution (20). In this case, the clinical team worked together to develop and customize the clinical evaluation tool to suit the third-year clinical students and faculty at UCQ. The process relied heavily on group collaboration to articulate the issues in clinical assessment and align the design of the tool to address those challenges. In this case, ambiguous terminology of competency attainment as presented in the CRNA ELCs and the lack of guidance and consensus in clinical assessment tools were identified as key issues.

Students, faculty and clinical buddy nurses were partners in the use of the new WCSP tool and were critical actors in the use and evaluation of it. Buddy nurses are nurses who work for the hospital and are responsible for training the nursing students by the bedside. Key characteristics of action research are the participatory nature and iterative, cyclical evaluation of the solution (20). As a team, the tool was holistically evaluated and customized as needed to suit particular instructional needs. Instructors were given the academic freedom to incorporate elements unique to their specific placement areas, allowing for flexibility while maintaining a consistent approach to student assessment. The tool was implemented during a defined period during the clinical course in the spring 2024 term. The use of the tool was observed, and feedback was collected through both formal and informal interactions with participants during meetings and discussions. The intention of piloting the tool was to complete the cycle of planning, developing, implementing and evaluating the tool, then define what would improve the tool. Though the pilot project did not yield formal qualitative or quantitative results, the formal use of the new WCSP tool produced good results in assessment as evidenced by positive feedback, appropriate use and voluntary applications to other courses.

### 2.1 Tool development

The concept for the WCSP tool was introduced by the Clinical Practice Lead, whose responsibilities included overseeing the placement of nursing students and clinical instructors. The role of the practice lead also involved ensuring the quality of clinical teaching meets best practice guidelines. Clinical evaluation has been a challenge for UCQ, and some of the authors, being members of the UCQ’s curriculum committee, initiated discussion of the challenges. The task of developing the tool was assigned to clinical course leads, the authors of this paper. Both course leads possess substantial experience in managing clinical courses and are well aware of the challenges involved in clinical teaching, particularly given the lack of clarity in competency expectations.

The development of the WCSP tool began with aligning course objectives to weekly expectations, as outlined in Table 1. The clinical learning was carefully scaffolded to support students’ progression in acquiring clinical competencies suitable for their level of study. In line with the concept-based curriculum, the tool integrated weekly concepts that were developed and implemented in the course. Feedback on the tool was actively solicited from other clinical instructors involved in the course throughout its development, from its initial conception to its eventual rollout. The Clinical Practice Lead also contributed feedback during this process. While the idea for the tool was in place for about 2 years, its creation and refinement took approximately 2 months. The fundamental aim of the tool was to establish weekly learning goals that progressively guided students toward the final objectives of the practicum. For example, by the end of the practicum, students are expected to independently care for at least two patients. The tool was designed to scaffold the skills required to achieve this goal, with a partial example provided in Table 1.

**Table 1:** Sample weekly clinical skills progress.

NURS 416 clinical placement weekly expected competencies			
Skills and competencies		Examples of instructor comments	Instructor monitor/tracker
Week 3	By week 3 patient focused assessments should be more comprehensive. Should be handling 1 patient. Able to demonstrate safe medication administration of minimum 4 classes of common medications. For example, Anticoagulants (Heparin, enoxaparin) Insulin (Aspart, Glargine) Beta Blockers, ARBS, PPI, ACEI, Pain medications- opioids and non opioids, NSAIDs Ability to recognize and discuss priorities based on endorsement and nursing assessments. For example, determining which patient to prioritize and identifying potential problems that could arise for each patient. Identify 2 key issues with each patient and be able to discuss them with buddy nurse. Students can explain the pre-operative, intra-operative, and post-operative processes. Students should be able to verbally explain the care of 1–2 patients from assessment through evaluation (using the nursing process) and demonstrate evidence of critical thinking and clinical judgment.	Issues with communication and developing professional and therapeutic relationships with patients.	Link to clinical tracker form
Week 4	Identify and promptly report abnormal findings, recommend interventions, and create a nursing care plan. Ability to perform assessments and vital signs on the buddy nurse’s patients. Work effectively with unit staff and healthcare professionals Increased competence in unit-specific skills, such as dressing changes and pre- and post-operative management.		Link to clinical tracker form

Clinical instructors were given the flexibility to implement the weekly concepts based on the learning opportunities available at their respective clinical sites. Throughout the development of the WCSP tool, feedback was actively sought from clinical instructors and the university’s Practice Lead to ensure its relevance and effectiveness. The tool also offered flexibility for faculty to adapt their teaching approaches by incorporating enhancements as needed. The tool was introduced to students during orientation and made available on the learning management system. Additionally, a printed version of the tool was distributed and explained to students on the first day of their clinical placement.

### 2.2 Participants and setting

This was not a formal research study as it aims to share the experience of the authors, clinical instructors, students, and buddy nurses implementing clinical assessment in a nursing program in Qatar. The study participants were both faculty and students of NURS416-Nursing Practice for High Acuity and Chronic Conditions. The total participants (students) included a third-year cohort composed of 87 male and female students aged between 20 and 35 years. Faculty members teaching this clinical course included Clinical instructors, Assistant Professors, and Associate Professors. These students were placed in multiple healthcare settings across public hospitals in Hamad Medical Corporation (HMC), Qatar, where they completed their medical-surgical clinical placements. The clinical environments included a range of acute and chronic care settings, providing the students with hands-on experience in treating adult patients admitted to medical and surgical units. The WCSP tool was implemented by the faculty teaching this course along with HMC buddy nurses in assessing the students enrolled in the course.

### 2.3 Implementation

Implementation of the WCSP tool began with introducing and explaining the tool to students in the clinical course. A thorough overview of the tool allowed students to review the clinical expectations on a week-to-week basis. Given that the buddy nurses at the clinical sites changed daily, students were responsible for communicating their daily learning objectives to their buddy nurses, as outlined by the tool. This approach aimed to keep both students and clinical staff aligned with the learning objectives set for the placement. As seen in Table 1, clinical instructors filled out their comments on the form every week and linked the clinical competency to the clinical tracker form.

## 3 Results and discussion

The implementation of the WCSP tool demonstrated positive outcomes, as evidenced by feedback from both instructors and students. The tool’s usefulness was attributed to its ability to facilitate instructors to continuously monitor and provide feedback on student progression. Many instructors reported that the WCSP document provided them with clear and concise goals, reminding them of what to focus on when monitoring students each week. The tool was particularly advantageous for the instructors teaching the course for the first time. For experienced instructors, the tool provided structure and guidance on a weekly basis, reinforcing its utility across varying levels of teaching experience. Instructors were surprised to see how much the WCSP tool provided guidance and clarity about the levels of competency achievement.

Feedback from students indicated that the WCSP tool was helpful in organizing their weekly activities and understanding weekly expectations. For stakeholders, including buddy nurses, the WCSP tool offered clarity and guidance, allowing them to provide appropriate support. For example, in the first 2 weeks, buddy nurses could focus on unit routines, communication, and nursing care plans rather than expecting students to take full responsibility for patient care.

Further, the WCSP tool provided evidence for identifying students who were falling behind, allowing faculty to adjust clinical teaching accordingly. Clinical supervision played a key role in helping students integrate theory into practice (21). A structured documentation of weekly expectations allowed both instructors and students to conceptualize defined learning objectives within clinical environments (17). The author further indicated that ongoing feedback in nursing education facilitated the learning process by offering students insight on current practice and practical advice for improvement. Previous research highlighted challenges related to the language and consistency of assessment tools used during nursing student clinical placements (22).

Some instructors who found success using the tool requested it for other courses. While no specific negative feedback on the WCSP tool was noted, it should be trialed and customized by other users, as this study only reflects the tool’s first iteration of development. The tool was recognized as flexible, providing a baseline for effective clinical assessment and customized to the clinical practice area. However, it may not apply to all clinical courses.

The WCSP tool ensured that all clinical instructors were aware of course expectations throughout the clinical rotations. The weekly clinical plan promoted consistency among students and educators across various healthcare facilities, enabling instructors to assess, evaluate, and track students’ progress. Furthermore, the document provided students with a clear understanding of the specific competencies they needed to achieve each week, with realistic and measurable goals. While the tool is still a work in progress, it shows promise in ensuring uniformity and success in attaining clinical competencies.

## 4 Recommendations

While the WCSP tool’s development is still in its infancy, positive feedback from students, faculty, and stakeholders indicated that it shows promise. It provided a clear roadmap for students to progress from basic to proficient levels in CRNA competencies by the end of the semester. However, this tool was trialed in a single clinical course within a specific cultural context. Its broader application in other clinical courses and nursing programs globally will require careful customization. This customization involves aligning the specific weekly indicators with the course objectives of each clinical course while considering cultural, institutional, and curricular variations across different nursing programs. Further discussion is required to explore how the tool can be customized and validated in diverse educational and cultural contexts to ensure its relevance and effectiveness.

The authors viewed the pilot launch as an initial step in the testing and evaluation process. The next steps involve identifying gaps, areas for improvement, and necessary modifications or customizations. Given that the tool was trialed in an accelerated semester, adjustments will be needed to adapt it for use in a regular-length semester. A potential limitation of this study is the anecdotal nature of the feedback collected from instructors and students. While the feedback provided valuable insights, it may have been subject to biases. Future research should include structured data collection and rigorous validation to strengthen the reliability and generalizability of the findings. Collaboration between clinical and academic colleagues is essential for the continued refinement of the tool, ensuring that nursing students are trained according to the competencies outlined by the standards while also aligning with expectations for clinical practicum. The tool should employ clear and specific language that all stakeholders can agree upon.

## 5 Conclusion

The use of the WCSP has provided clear and specific goals for the students and the instructors to work with, thereby adding consistency to clinical evaluations in one clinical course at UCQ. This highlights the ongoing need for the development of clinical assessment tools that are tailored to the level of study and the clinical setting. Wu et al. (23) in their systematic review of clinical assessment had suggested that the tool should be able to capture multiple dimensions of learning in the clinical environment and should involve all the stakeholders. Almakawi et al. (24) stated that universal nursing competencies, especially in the present climate where the migration of the nursing workforce is visible, and countries face persistent challenges in assessing competencies, should be standardized globally. Significant work remains to be done in the field of clinical assessment, and collaboration among nursing educators will be crucial in addressing these challenges. The authors of this paper call for more investment of time, effort and global collaboration to advance the development of effective clinical assessment tools that meet the needs of an evolving healthcare landscape.

## Data Availability

The raw data supporting the conclusions of this article will be made available by the authors, without undue reservation.
